# A pilot exploratory investigation on pregnant women’s views regarding STan fetal monitoring technology

**DOI:** 10.1186/s12884-017-1598-8

**Published:** 2017-12-29

**Authors:** Kate Bryson, Chris Wilkinson, Sabrina Kuah, Geoff Matthews, Deborah Turnbull

**Affiliations:** 1 University of Adelaide, School of Psychology, Adelaide, South Australia Australia; 2 Women’s and Children’s Health Network, Adelaide, South Australia Australia

**Keywords:** Labour, Fetal monitoring, Intrapartum care, Maternity service providers, Qualitative research

## Abstract

**Background:**

Women’s views are critical for informing the planning and delivery of maternity care services. ST segment analysis (STan) is a promising method to more accurately detect when unborn babies are at risk of brain damage or death during labour that is being trialled for the first time in Australia. This is the first study to examine women’s views about STan monitoring in this context.

**Methods:**

Semi-structured interviews were conducted with pregnant women recruited across a range of clinical locations at the study hospital. The interviews included hypothetical scenarios to assess women’s prospective views about STan monitoring (as an adjunct to cardiotocography, (CTG)) compared to the existing fetal monitoring method of CTG alone. This article describes findings from an inductive and descriptive thematic analysis.

**Results:**

Most women preferred the existing fetal monitoring method compared to STan monitoring; women’s decision-making was multifaceted. Analysis yielded four themes relating to women’s views towards fetal monitoring in labour: a) risk and labour b) mobility in labour c) autonomy and choice in labour d) trust in maternity care providers.

**Conclusions:**

Findings suggest that women’s views towards CTG and STan monitoring are multifaceted, and appear to be influenced by individual labour preferences and the information being received and understood. This underlies the importance of clear communication between maternity care providers and women about technology use in intrapartum care. This research is now being used to inform the implementation of the first properly powered Australian randomised trial comparing STan and CTG monitoring.

**Electronic supplementary material:**

The online version of this article (10.1186/s12884-017-1598-8) contains supplementary material, which is available to authorized users.

## Background

Australian health policy recommends the incorporation of maternity consumers’ views to inform planning and development of services [1]. Women’s views, including their thoughts, opinions, and preferences towards aspects of care, carry important implications for postnatal psychological functioning [2] as well as for decisions by maternity care providers [3, 4].

In an Australian-first research initiative, researchers at a publically funded maternity hospital in the state of South Australia conducted a pilot randomised controlled trial (RCT) comparing STan monitoring (supplementing concurrently performed CTG) compared to CTG monitoring alone with the aim of determining if STan could reduce emergency caesarean section rates [5] and other interventions, whilst maintaining or improving safety of the unborn baby [6]. While some international evidence at the time did not support such a hypothesis [7–9], much of the existing research emanated from vastly different clinical environments [7, 9] and the extent to which such research was thought to be generalisable to an Australian context at the time of the current research, was arguable.

Cardiotocography (CTG) for fetal monitoring during labour is usually recommended if there are concerns that a pregnant woman’s unborn baby is less likely to cope with the stress of labour, or if a woman requires augmentation with oxytocin or elects to have an epidural for pain relief [10]. STan is a method of intrapartum fetal monitoring, which provides clinicians with additional physiological information to that provided by the CTG about how the unborn baby is responding to the stress of labour [6]. STan monitoring may enhance detection of intrapartum fetal distress [6], possibly reducing false positive diagnosis of fetal distress which has, in turn, been described as possibly associated with CTG monitoring [8, 11]. False positive diagnosis of fetal distress can, in turn, result in excessive obstetric intervention. Over-diagnosis of fetal distress may be a contributing factor to wide variation in caesarean sections rates in many countries including Australia [12].

We have identified one quantitative study that has investigated women’s retrospective self-reported satisfaction with STan monitoring in a U.K. hospital, in which the majority of women viewed STan as an acceptable intrapartum intervention [13]. While such studies can be useful in evaluating services, they may have limited ability to identify the factors that can shape women’s views about aspects of their care [4, 14, 15]. This knowledge gap requires urgent attention in light of differences between CTG and STan monitoring, briefly discussed below.

In CTG monitoring, information about the fetal heart rate and mother’s contractions are usually recorded by a monitor connected to a belt worn over the woman’s abdomen [8, 11]. In about 10% of cases, CTG may require use of an internally inserted electrode that is attached to the unborn baby’s scalp [8, 11]. Unlike CTG monitoring, STan, being a system that analyses the electric currents from the baby’s heart, the fetal electrocardiographic waveform (the ECG), will always require the use of a scalp clip [6]. Research on women’s labour and birth preferences has identified mobility as a labour preference [16, 17]; however little research exists about women’s views towards STan in the context of such preferences. Such research would prove useful given that intrapartum fetal monitoring is a common aspect of Australian maternity care practice [18]. Therefore, the aim of this pilot, exploratory investigation was to develop an understanding of pregnant women’s prospective views towards STan (plus CTG monitoring) in comparison to CTG monitoring alone. The investigation was conducted alongside the pilot randomised trial to contribute knowledge about aspects of fetal monitoring important to women’s views so as to inform a subsequent discrete choice study.

## Methods

### Participants

Recruitment was carried out at the Women’s and Children’s, a tertiary maternity unit hospital which manages the largest number of births in the state of South Australia.

After considering selected research about the factors that may affect women’s views of labour [17, 19, 20] maximum variation sampling was used to recruit across parity, age, maternity care setting, and pregnancy gestation (Table 1) [21].

**Table 1:** Participant characteristics

*Characteristics*	*Sample (n = 11)*
Age range (years)^a^	
19–22	1
23–26	3
27–30	1
31–34	5
35–38	1
Parity	
Nulliparous	7
Parous	4
Gestation (weeks)^b^	
≤ 14	1
15–19	1
≥ 20	9
Maternity care setting	
Antenatal Clinic	1
Caseload Midwifery	4
Maternal Fetal Medicine Clinic	6

^a^Participant age presented as age range to preserve anonymity

^b^Pregnancy gestation in weeks based on perinatal statistical guidelines [18]

The approach was supplemented by a theoretical sampling frame to ensure that the recruitment included locations where low and higher risk pregnancies were managed. For instance, Caseload Midwifery usually manages lower-risk pregnancies and makes referrals to obstetric care as required. Pregnancies that may be complicated by maternal and/or fetal risk factors are usually cared for in obstetrician-led care settings including generalist Obstetric Antenatal and subspecialty Maternal Fetal Medicine clinics.

In order to be eligible, participants had to be: over 18 years old; a minimum of 12 weeks pregnant; currently receiving hospital based care; and have sufficient written and spoken English proficiency. They also had to be considered by their referring clinician as being unlikely to experience distress as a result of discussing a situation of labour and childbirth. Because STan aims to reduce false positive detection of fetal distress requiring operative intervention, thereby theoretically increasing a woman’s chance of attempting a vaginal delivery where it is safe to do so, the study aimed to interview women for whom a vaginal birth was a preferred and appropriate option. This meant that women with previous caesarean section history were excluded given that vaginal birth after caesarean section is not always recommended [22].

### Recruitment

Human Research ethics approval was gained on 10 July 2015 from both hospital and university review committees. The first author (hereafter referred to as researcher) consulted midwives and clinicians across different maternity care locations to discuss research aims and recruitment. Eligible women were identified and informed about the study by midwives or clinicians. The researcher contacted women who had provided informed consent to discuss research participation, and interviews were arranged. Interviews were conducted in the hospital or off-site at a café. Participants received complimentary food and/or drink at the hospital café.

### Data collection

From August–October 2015, the researcher carried out semi-structured interviews lasting 10–30 min, which were audiotaped with participants’ informed consent. The interview guide included two written hypothetical scenarios (Additional file 1), one depicting CTG monitoring and the other, STan monitoring. The scenarios were developed by the research team, which included several clinicians. They aimed to prompt discussion and communicate sufficient information so that women could compare and contrast CTG and STan monitoring. Table 2 summarises the main features of the scenarios, which were written in first person to encourage the participant to suggest hypothetical, prospective preferences [23]. They were matched for reading age, and were piloted for comparable word length and medical content to enhance qualitative rigor [24].

**Table 2:** Participant scenario variables

*Variables compared across scenarios*	*CTG scenario*	*STan scenario*
External fetal monitoring method	Two discs (transducers) rest on mother’s stomach, one disc measures heartbeat and other disc monitors contractions.	Not available.
Internal fetal monitoring method.	Electrode inserted through woman’s vagina, and attached to unborn baby’s scalp. Lead is attached to mother’s thigh.	Only fetal scalp electrode can be used. Electrode inserted through woman’s vagina, and attached to unborn baby’s scalp. Lead is attached to mother’s thigh.
Movement during labour and contractions.	Abdominal belt: can stand, and move within a two-metre radius (length of cord connecting woman to monitor). Or, if using scalp electrode, must lay flat on bed.	Restricted mobility because need to be connected up to STan monitor. Mostly confined to bed.
Delivery associated with each technology.	Increased chance of operative intervention including caesarean section.	Decreased chance of operative intervention including caesarean section.

Participants were encouraged to ask questions to clarify their understanding before being asked their views about CTG and STan. They were asked four open-ended questions informed by guidelines for qualitative interviewing in healthcare [24].

The researcher introduced herself as a postgraduate psychology student, with no clinical or organisational association with the hospital. Birth and labour are acknowledged as sensitive interview topics [25], and the researcher used practical strategies described in the qualitative interviewing literature to promote an environment in which participants may comfortably express their views [26]. For instance, participants were encouraged to be accompanied by a supportive partner, and the researcher reflected back to the participant during interviews, to check her understanding of the participant’s talk. The researcher maintained contact with midwives and obstetricians throughout recruitment.

The researcher transcribed all interview recordings. Participants were recruited continuously and purposively alongside data analysis, in keeping with qualitative research design [21, 24]. This enabled the researcher to theoretically sample across three different maternity care locations (Caseload Midwifery, Maternal Fetal Medicine Clinic and Antenatal Clinic) in the one hospital.

### Analysis

Analysis was influenced by a realist epistemology to describe the social phenomenon (women’s views) from participants’ perspectives [27], so that results could be presented in an applied healthcare format intended for a broad readership [28]. Transcripts were analysed using a descriptive and interpretative thematic analysis method [27]. Word-processing software was used to systematically and openly code segments of transcripts that appeared relevant to the research aim [27]. Two transcripts coded by the researcher were crosschecked for accuracy by the last author at two separate time points, and both the researcher and last author communicated regularly to discuss codes and analytic decision-making, as documented in an audit trail produced by the researcher [27, 29]. Development and application of codes to the transcripts were documented in a codebook by the researcher [30]. Codes were arranged into provisional, interrelated themes considered representative of patterns across the dataset in relation to the research aim [27] and the researcher used manually developed data saturation tables to monitor prevalence of themes in the dataset [31]. Saturation of themes was determined after the eleventh interview; because the most recently conducted interviews appeared to yield no new themes [30, 31].

#### Results

The dataset consisted of 11 transcribed interviews with participants from: the Antenatal Clinic, Maternal Fetal Medicine Clinic and Caseload Midwifery. Most participants expressed a preference for CTG monitoring compared to STan monitoring. Based on analysis and interpretation of transcripts, four key themes are described: a) risk and labour b) mobility in labour c) autonomy and choice in labour d) trust in maternity care providers. While women’s responses were often quite generic and seemingly unrelated to one type of monitoring or another, we have aimed to highlight responses that show the comparison from the women’s perspectives, of the two monitoring methods.

### Risk and labour

The majority of participants’ talk about fetal monitoring was framed by perceptions of labour and childbirth as uncertain and somewhat risky events. Women who were able to experience uncomplicated and straightforward labours were often described as “lucky” [03, 23–26, parous, maternal fetal medicine] exceptions. Compared to parous participants, nulliparous participants emphasised the perceived uncertainties of labour:

…‘cos you never know [what] labour might be like (.) we all plan for you know ah good ah uncomplicated but you never know

[010, 31–34, nulliparous, caseload midwifery].

Some participants from Caseload Midwifery described maternity care as a “medicalised environment” [011, 31–34, nulliparous, caseload midwifery], involving intervention to deliver babies within a short time frame:

…like I obviously I understand though that now everything is a lot more cautious and like careful like women don’t just have babies

[01, 23–26, nulliparous, caseload midwifery].

Some participants from Caseload Midwifery also expressed concerns about what they perceived as a high Australian caesarean rate.

Complicated labour resulting in negative childbirth experiences were in turn perceived as a risk to postnatal maternal wellbeing, and discussed by participants in terms of impacting on future maternal outcomes:

I don’t want to feel like I never want to do that okay ((laughs)) like I don’t want to have anxiety about like you know ever having to have another baby ‘cos if you had like a traumatic birth

[01, 23–26, nulliparous, caseload midwifery].

Intrapartum fetal monitoring was discussed as a risk management strategy; monitoring was perceived in terms of both reassurance and unease. Regardless of CTG or STan, fetal monitoring was perceived as a method of medical precaution:

If, so I think I would have gone for monitor it’s never gonna hurt it’s just there….yeah it’s the reassurance

[010, 31–34, nulliparous, caseload midwifery].

By contrast, other participants expressed that talking about fetal monitoring, regardless of method, was “scary” [04, 19–22, nulliparous, maternal fetal medicine], exemplified in the following interaction:

I: What is your immediate response to reading the two scenarios ((pause)) what most catches your eye?

P: Um they sound fairly similar um I would be apprehensive about having either

[06, 31-34, nulliparous, maternal fetal medicine]

Compared to CTG monitoring, STan was generally viewed as more appropriate management for complicated pregnancies, because it was perceived to more closely monitor the unborn baby. One participant suggested that compared to CTG, STan monitoring “gives you more results so it measures like everything, more things in your child” [03, 23–26, parous, maternal fetal medicine].

Some participants also talked about the need to use STan monitoring judiciously, for example in intrapartum situations in which the risk status of the unborn baby was already medically ascertained:

So if it was a medical background there or medical reasons to have that additional monitoring then I would feel like it’s probably justified if it was just a everyone gets stuck to it I would probably think that it’s not necessary

[011, 31-34, nulliparous, caseload midwifery]

By contrast, another participant supported use of STan monitoring at the outset of labour, regardless of perceived pregnancy risk:

Yeah I guess like this one [STan] in theory ‘kinda seems like better in the sense that it gives you a better idea of like if your baby is stressed

[01, 23–26, nulliparous, caseload midwifery].

Talk about uncertainty and risk seemed to frame participants’ discussion about use of the fetal scalp clip. Whereas participants generally accepted CTG using an abdominal belt, the fetal scalp clip that is sometimes used in CTG monitoring but required by STan monitoring, was generally less supported:

The CTG where it’s monitored on your stomach is fine but the clip attached to your baby’s head just for me personally is ((laughs)) weird

[05, 31-34, parous, maternal fetal medicine]

Perceived apprehension towards use of the scalp clip reflected uncertainty about internal monitoring:

Yeah it’s very scary sounding.

[09, 23–26, nulliparous, maternal fetal medicine]

I’m not sure whether there would be particular risks associated with that it just seems a bit more invasive than might be necessary

[011, 31–34, nulliparous, caseload midwifery].

By contrast, another participant perceived the scalp clip as less invasive, relative to other procedures that she had experienced in previous care:

You know you get examined so much it’s a standard thing when you get pregnant so this STan monitoring wouldn’t affect me in an invasive form

[03, 23–26, parous, maternal fetal medicine].

Overall, participants associated the scalp clip with perceived pros and cons and expressed a need to carefully consider these in the pursuit of a better outcome for the unborn baby:

Um one of the pros if there’s more information I guess that you get from going and putting the clip in like that ((pause)) it probably be something to look in to ((pause)) like if it gave you more information not sure what but yeah ((pause)) and I guess the other side would be like it’s pretty intrusive it’s pretty ((pause)) you’d wanna make sure that it’s safe while sticking the clip in and placing it

[02, 35–38, parous, maternal fetal medicine].

Views about risk possibly associated with STan were further reflected in discussion about it being a new technology in Australia. On the one hand, STan seemed to be associated with improved monitoring performance:

...it’s a new technology so it might be able to get more information.

[06, 31–34, nulliparous, maternal fetal medicine].

On the other hand, concerns were raised about STan being an “untested” technology [02, 35–38, parous, maternal fetal medicine], and apprehension surrounding new technology seemed to frame some participants’ views:

Have they even used this before?…nah I think I would be sticking to the CTG just because I guess this [STan monitoring] is new and trying something new probably for the first labour might be a little bit crazy

[09, 23–26, nulliparous, maternal fetal medicine].

Apprehension towards a new technology was sometimes juxtaposed with expressed uncertainty towards existing fetal monitoring practice. This is reflected in one participant’s talk about her previous experience of CTG monitoring:

…like the baby moves and then it looses track of where the heart rate is with the CTG and I also have noticed a couple of times when I’ve had contractions the CTG doesn’t pick it up

[07, 27–30, nulliparous, antenatal clinic].

### Mobility in labour

Participants from the Antenatal Clinic, Maternal Fetal Medicine Clinic and Caseload Midwifery discussed mobility in ways that suggested it as a multifaceted labour preference. This preference was largely supported by perceptions from both parous and nulliparous participants that mobility and adjusting body positions are beneficial pain management techniques in labour. Some participants discussed that they “wouldn’t hesitate” [08, 31–34, parous, caseload midwifery] to adjust their mobility preferences in favour of being connected to STan, in the pursuit of what they perceived as improved fetal outcomes. For example, one participant identified that the “biggest thing with the STan monitoring is that you can’t get up and walk around”. This participant later discussed adjusting this preference in favour of STan monitoring, in a situation of perceived medical need:

You’d choose your baby’s health over you being comfortable any day…most mothers make that decision anyway

[03, 23–26, parous, maternal fetal medicine].

This participant previously talked about how she did not get the opportunity to move around in her previous pregnancy. In addition, some participants discussed the importance of mobility in terms of fulfilling other important labour preferences, including the ability to “..get in and out of the bath” [07, 27–30, nulliparous, antenatal clinic].

### Autonomy and choice in labour

Preferences towards CTG or STan monitoring were described by participants as one of the many choices expected of pregnant women. Participants talked about labour as a “really personal thing” [011, 31–34, nulliparous, caseload midwifery], and unique to every woman. Consequently, observed hesitancy appeared to underlie responses to being asked to recommend a monitoring preference to a pregnant friend:

I’m not one that gives opinions like that very often like I’d probably tell them about both and say what my choice was but yeah I’d obviously let them make their own decision

[08, 31–34, parous, caseload midwifery].

A reoccurring preference from participants was for labour to be as “natural as possible” [011, 31–34, nulliparous, caseload midwifery]; however there appeared a range of views regarding STan monitoring in the context of what was perceived to be ‘natural’ birth. Compared to CTG, STan appeared to conflict with perceptions of a natural birth:

This one [STan] was more invasive in the process that it’s inserted and it’s actually attached to the baby so I don’t know my point of view I’d like it all to be natural so at that stage when this little being’s coming into the world

[08, 31–34, parous, caseload midwifery].

By contrast, another participant discussed STan as a method of facilitating a vaginal birth. This participant expressed concern about the caesarean section rate resulting from premature risk assessment of the unborn baby. STan was discussed as a method to possibly mitigate unnecessary intrapartum interventions, and this is reflected in the following excerpt taken participant discussion about perceived pros and cons of CTG and STan:

I think probably a negative of this one [CTG] is like you’d be in a rush to get your baby out…and there’s a more like a chance of like forceps and suction cups (.) like all the things that you don’t want ideally in the end ((laughs)) and I guess with that one [STan] you think they will slow your labour down and like give you a bit more of a chance to have things happen more naturally yeah less intervention

[01, 23–26, nulliparous, caseload midwifery].

The importance of encouraging and respecting maternal choices in labour was widely discussed by the majority of participants. The expectation for increased choice towards labour and birth preferences was also described as somewhat overwhelming:

I guess no one really does talk about it to you get closer ‘til you get closer to the labour…that’s when they go through all the choices and you don’t know which one to do

[04, 19–22, nulliparous, maternal fetal medicine].

Moreover, some participants talked about the practical limits to maternal preferences for labour, because they could be subject to change unexpectedly:

You can’t make plans babies don’t stick to a plan… they do their own thing

[07, 27–30, nulliparous, antenatal clinic].

Another participant discussed that while she valued doing “a bit of research” about decisions she would need to make in labour, including fetal monitoring, she acknowledged that she may not be able to follow-through with her initial preferences in a situation of actual labour, during which a woman is “in pain and emotionally a bit all over the place” [011, 31–34, nulliparous, caseload midwifery].

### Trust in maternity care providers

This final theme describes the perceived importance of monitoring recommendations from doctors and midwives. Four participants in the sample expressed difficulty in making a hypothetical preference for CTG or STan monitoring, choosing instead to accept recommendations from doctors and midwives. Perceived trust in doctors’ monitoring recommendations was discussed as an important factor to participants from the Maternal Fetal Medicine clinic:

I’m a very doctors tell me to do something do that…they’re the ones with years of experience.

[03, 23–26, parous, maternal fetal medicine].

Participants from Caseload Midwifery discussed the importance of communicating their intrapartum preferences and values about birth to their midwives, who participants perceived would, in turn, recommend a suitable monitoring method. Some participants from Caseload Midwifery discussed the influence of doctors’ and midwives’ views on intrapartum care:

It’s also about like being trusting with people that are working with you and trusting that they’re gonna take the right information from the monitoring and do like what’s best, ‘cos in my opinion there’s always gonna be doctors and midwives who are more inclined to say okay let’s intervene than ones that won’t

[01, 23–26, nulliparous, caseload midwifery].

## Discussion

This study examines for the first time, the views of Australian women about two different forms of fetal monitoring. The study was conducted alongside a pilot randomised trial and provides insights, which will ultimately be used to develop a discrete choice experiment, which will examine the trade offs that women would be prepared to make to achieve desired outcomes. Since this study was performed, a pilot trial has found that a reduction in emergency caesarean section may be clinically plausible within an Australian context [5], and consequently, an appropriately powered randomised trial has commenced.

Strengths of the current study include its on-going, theoretical sampling strategy [21] to recruit a varied and heterogeneous group of maternity consumers in Australia [1]. Further strengths include the interview guide informed by qualitative interviewing guidelines, and emphasis on maintaining a comfortable interviewing environment to elicit participants’ discussion [24, 26].

The finding that participants perceived STan monitoring as an improved fetal monitoring method, but mainly for complicated pregnancies and/or in instances in which the unborn baby is compromised, was similarly reported in the previous study on women’s satisfaction of STan [13]. Pregnancy risk factors can be difficult to ascertain from labour outset and degree of both perceived and actual pregnancy risk may change [32]. Analyses carry implications for maternity care providers’ communication of STan to consumers; specifically, how STan is intended to alert clinicians to problems in labour before they escalate, rather than respond to problems after they become emergencies [6]. This may be important given results of a previous study that reported variable accuracy in the content of consumer-focused maternity information about fetal monitoring [33].

Analyses that fetal monitoring, irrespective of CTG or STan, was perceived with both reassurance and unease were similarly reported in a previous qualitative study comparing women’s views towards two methods of CTG, which made no reference to the use of a fetal scalp clip [34]. In comparison, our study found that some participants seemed to perceive the scalp clip as contributing additional uncertainty to the unpredictable and sometimes risky event of labour. Analysis of varied participant concerns towards the scalp clip underlies the need for consumer information to explain factual risks involved with the procedure, to enable decision-making that is aligned with women’s views and care preferences. This seems important given that some participants appeared to consider use of the scalp clip as a somewhat reluctant but acceptable trade-off in the pursuit of what they perceived as improved fetal outcomes. This nuanced perception does not appear to be captured in existing literature on STan monitoring [13]. This, in turn, can speak to the need for care providers to consider women’s views towards their care, rather than make assumptions, when determining approaches to care delivery [4].

When making decisions about intrapartum care, women have been described as seeking to minimise risks alongside promoting their values and ideals for birth [25]. Most participants in the current study preferred a ‘normal and/or natural’ vaginal birth. There was variation in participants’ views towards STan in the context of a ‘natural’ birth, especially from participants within the same care setting (Caseload Midwifery). This finding may be consistent with previous research suggesting that that the term ‘natural’, while often associated with minimal intervention, can be thought of as multidimensional in nature [35].

It has been described that women prefer to have control of, and increased choice about aspects of intrapartum care [1, 4]. The current analyses suggest that some participants preferred to accept the monitoring method recommended by their maternity care providers. This may be consistent with research findings describing that a woman may also perceive control through delegating decision-making about aspects of maternity care to her medical caregivers [25, 35]. This highlights the need for maternity care providers not assuming that all women prefer to be involved in the same way in intrapartum decision-making [4, 35].

Current analyses suggested nuances in participants’ views towards doctors/midwives’ recommendations about fetal monitoring. Caseload Midwifery participants discussed the importance of articulating their values and preferences to their midwife who, in turn, could make the most appropriate recommendation aligned with participants’ values. Participants from the Maternal Fetal Medicine Clinic and Antenatal Clinic expressed trust in their doctors’ recommendation for labour management. These analyses may, with reference to the literature, reflect the possible influences of the maternity care service provided to women at the time, as well as information and guidance provided by maternity caregivers on women’s views towards intrapartum care provided to them [13, 19, 36].

Current analyses that participants seemed to perceive physiological benefits from mobility in labour as opposed to being confined to bed are described in the literature [37]. Some participants seemed to reconsider their preference for mobility in favour of using STan in perceived medical need, and for closer monitoring of the unborn baby. This seems consistent with the emerging body of literature on the complexity of women’s intrapartum preferences, and how they can vary in response to other care preferences as well as a woman’s experience of mobility in her previous labour [17]. Current findings must be considered in light of the described pressure on women to sometimes adjust maternity preferences, including mobility, in favour of prioritising their unborn baby’s wellbeing in labour [25]. This highlights a need for consumer information to clearly explain the impact of fetal monitoring on women’s mobility in labour. Future research could investigate women’s views towards updated versions of STan with a wireless option that is potentially less restrictive to women’s mobility.

Analyses that STan was perceived as somewhat risky because it is a new technology may support findings from a previous study that investigated women’s preferences for aspects of maternity care provision [19]. That study reported the issue of potential bias of women favouring the existing maternity care service provided to them, which may impact on consumer uptake of change in maternity practice [19].

Analyses of women’s reluctance to recommend either CTG or STan to a pregnant friend may align with previously described understandings of labour and childbirth as individualised events over which women prefer to exercise choice. Selected literature has suggested that the expectation on women to make autonomous decisions and choices towards their preferred labour and birth experiences may carry problematic implications for women [38]. It has been described that the ability to exercise autonomous decision-making over the labour and birth experiences women want, may not be available to all women, given the somewhat unpredictable nature of labour and birth and variability in pregnancy risk [38, 39], and inconsistent research of the effects on posture on labour outcomes for nulliparous women [40] Some participants in the current study doubted their ability to make intrapartum decisions during “full-blown labour”.

Limitations requiring consideration when interpreting findings include the somewhat narrow participant recruitment from the Maternal Fetal Medicine clinic. This may have implications for interpretation of participants’ views towards STan monitoring, as type of maternity care service is acknowledged as a possible factor shaping women’s opinions about care offered to them [19]. Another limitation is the relative brevity of some of the interviews, which was especially the case for those conducted in the Maternal Fetal Medicine Clinic. In-depth exploration of participants’ views towards, among other topics, sources of maternity care information, perceived risk to the unborn baby in labour and the value of intervention in childbirth were beyond the scope of the current study.

Another limitation warranting consideration when interpreting the current findings is the influence of the local setting on the content of the participant scenarios. Content about the key aspects of CTG and STan, including the comparisons about operative delivery rates, was based on assumptions by the team at the time about the potential for STan to result in reduced emergency caesarean section rates, rather than the extant research evidence [7–9].

Further, the interview scenarios described CTG and STan as separate conditions, rather than the condition of STan monitoring (in addition to CTG) compared to the condition of CTG alone. While this decision intended to maximise simplicity and provide an opportunity to discuss key differences between the two methods, it is recognised that the interview scenarios describe conditions that differ from those tested in the pilot randomised trial conducted at the public hospital at the time.

By its very nature, the study included only women who were open to talking about their views on care. It was not possible to determine the number of potential participants who declined to participate after being initially identified by clinicians as eligible. Significant exclusion criteria prevented inclusion of participants from different cultural backgrounds as was done in the one previous study on maternal satisfaction towards STan [13]. Cultural factors are considered important to women’s maternity care views [4] and this could be explored in future research on women’s views towards monitoring.

## Conclusions

Analyses from this pilot exploratory investigation suggest that women’s views towards use of fetal monitoring as part of intrapartum care may be influenced by factors including perceived risk to the unborn baby in labour, mobility in labour, autonomy and choice, and trust in recommendations from medical caregivers. These findings may be used to inform the development of consumer information to best support women to make informed and values-based choices about monitoring methods in labour. Second, they might be used to assist the development of attributes for future discrete choice studies for inclusion in future economic analyses of fetal monitoring.

## Additional file

Additional file 1: Participant Interview Scenarios. (PDF 144 kb)

## Abbreviations

CTG: Cardiotocography
ECG: fetal electrocardiographic waveform
STan: ST segment analysis
