# Supplementary material for: A pilot exploratory investigation on pregnant women’s views regarding STan fetal monitoring technology
Source: BMC Pregnancy Childbirth. 2017 Dec 29;17:446. doi: 10.1186/s12884-017-1598-8 (PMC5747096; doi:10.1186/s12884-017-1598-8)
Supplement: Additional file 1: — Participant Interview Scenarios. (PDF 144 kb) [file 12884_2017_1598_MOESM1_ESM.pdf]

Scenario 1

Your baby will be monitored during labour by a machine called a CTG. CTG records a baby's heart rate pattern in labour. Your medical team of doctors and midwives will look at the heart rate to decide if you need extra help to deliver your baby.

To set-up the CTG, you can lay upright on the bed while gel is put on your belly. Two tea saucer-sized plates are attached on your belly by an elastic belt. One plate records the baby's heart rate. The other monitors your contractions. You are connected to the machine by a two metre long cord. You may sit and stand while wearing the monitoring belt, and wear it until your baby is born.

Sometimes, the baby's heart rate is recorded in a different way. A small clip (the size of your little finger nail) is attached to your baby's head. The clip is inserted into your vagina and up towards your baby's head using a thin cord. The remaining cord is connected to a patch on your leg. This patch is held in place by a strap.

You will be connected to the CTG in this way until your baby is born. The small clip will be removed just before the baby is born.

If the CTG records a concerning heart rate, then your medical team will help you have your baby within a short time. This may mean that your doctor will use a suction cup on your baby's head or forceps to ease your baby out.

As the last resort, you may be prepared for a caesarean. Afterwards it may be found by the medical team that a caesarean section wasn't needed.

Scenario 2

Your baby will be monitored during labour by a machine called STAN. STAN records specific information about what is happening to your baby's heart rate and oxygen supply in labour. Your medical team of doctors and midwives will use this information to decide if you need extra help to deliver your baby.

During labour, you are always connected to STAN by lying flat or sitting on the bed. A thin cord connected to the monitor is strapped to a patch on your leg. A small clip about the size of your little fingernail is attached on the end of the cord. The cord is inserted into your vagina, and up towards your baby's head, where the clip is attached.

You will be connected to STAN in this way until your baby is born. You might not be able to stand or walk around much during your contractions. The small clip attached to your unborn baby's head will be removed just before it's born.

Any complications with your baby are recorded as a 'ST event'. If a ST event occurs before you start pushing, then the midwives will help you to adjust your position. They may also increase your fluids and help you to slow down your contractions.

If a ST event occurs when you are pushing, then the medical team will help you have your baby within a short time. This may mean that your doctor will use a suction cup on your baby's head or forceps to ease your baby out. As a last resort, you may be prepared for a caesarean.
